# Salt Stress Leads to Morphological and Transcriptional Changes in Roots of Pumpkins (*Cucurbita* spp.)

**DOI:** 10.3390/plants14111674

**Published:** 2025-05-30

**Authors:** Hongjiu Liu, Ding Ding, Yeshuo Sun, Ruiping Ma, Xiaoqing Yang, Jie Liu, Guoxin Zhang

**Affiliations:** 1 Institute of Coastal Agriculture, Hebei Academy of Agriculture and Forestry Sciences, Tangshan 063299, China; liured9@nwsuaf.edu.cn (H.L.); dddyiman@163.com (D.D.); sys19930317@163.com (Y.S.); marp0825@126.com (R.M.); yxqing708@163.com (X.Y.); 2 Dong Fang Chia Tai Seed Co., Ltd., Beijing 100176, China; 13910578696@163.com

**Keywords:** NaCl concentration, pumpkin, root architecture, transcriptomic profile

## Abstract

Salinity stress poses a major challenge to agricultural productivity worldwide, including for pumpkin, a globally cultivated vegetable crop with great economic value. To deal with salt stress, plants exhibit an array of responses such as changes in their root system architecture. However, the root phenotype and gene expression of pumpkin in response to different concentrations of NaCl remains unclear. To this end, this study evaluated the effects of salinity stress on root architecture in *C*. *moschata* (Cmo-1, Cmo-2 and Cmo-3) and *C*. *maxima* (Cma-1, Cma-2 and Cma-3), as well as their hybrids of *C*. *moschata* and *C*. *maxima* (Ch-1, Ch-2 and Ch-3) at the germination and seedling stages. The results showed that the total root length and the number of root tips decreased by more than 10% and 5%, respectively, under 180 mM NaCl conditions compared to those under the 0 mM NaCl conditions. In contrast, the total root length and the number of root tips were increased or decreased under 60 mM NaCl conditions. Meanwhile, salt stress was considered severe when treated with more than 120 mM NaCl, which could be used to evaluate the salt tolerance of the germplasm resources of pumpkin. In addition, the transcriptional changes in the roots of both Cmo-3 and Cma-2 under salt stress were analyzed via RNA-sequencing. We found 4299 and 2141 differential expression genes (DEGs) in Cmo-3 and Cma-2, respectively. Plant hormone signal transduction, Phenylpropanoid biosynthesis and the MAPK signaling pathway were found to be the significant KEGG pathways. The expression of *ARF* (*auxin response factor*), *B-ARR* (*type-B response regulator*) and *PYR* (*pyrabactin resistance*)/*PYL* (*PYR-LIKE*) genes was downregulated by NaCl treatment. In contrast, the expression of *SnRK2* (*sucrose non-fermenting-1-related protein kinase 2*) and *AHP* (*histidine-containing phosphotransmitter*) genes was downregulated in Cmo-3 and upregulated in Cma-2. These findings will help us better understand the mechanisms of salt tolerance in pumpkins and potentially provide insight into enhancing salt tolerance in crop plants.

## 1. Introduction

Salt stress is one of the most significant environmental factors reducing yield in arid and semi-arid areas, posing a direct threat to global food resources [1]. Plants exhibit notable changes in root architecture in response to salt [2]. It is well-known that salt stress not only inhibit both primary root and lateral root growth in *Arabidopsis*, rice and soybean [3,4], but also significantly affect root architecture in the maize, wheat and apple [5,6,7]. Meanwhile, lower NaCl levels stimulate lateral root formation [8]. Many studies have described the effect of the application of different concentrations of NaCl on root growth at the level of histological anatomy, plant physiology and molecular biology [9,10,11,12]. Based on the growth of different types of roots in *Arabidopsis*, *Casuarina equisetifolia*, Indian mustard, and triticale [10,13,14,15], the dose of NaCl was screened in the previous studies, which provided guidance on NaCl concentration for salt-tolerance mechanism analyses and breeding salt tolerant varieties. However, in pumpkins, the concentrations of NaCl corresponding to mild and severe salt stress are still unknown, which represents an obstacle to salt-tolerance germplasm resource identification in pumpkins. Additionally, the increases or decreases in the root length and number of tips under NaCl treatment at the germination and seedling stages also remain unclear.

Pumpkins belong to the genus *Cucurbita*, one of the most diverse genera in the Cucurbitaceae family, which includes several widely cultivated crop species [16]. *Cucurbita moschata* (*C. moschata*), *Cucurbita pepo* and *Cucurbita maxima* (*C. maxima*) are widely used for human consumption, as fodder for livestock and for ornamentation [17]. In addition, *C. moschata*, *C. Maxima* and their hybrids have been widely used as rootstock for cucumber, melon, and watermelon to gain higher yields and fruit quality [18]. Overall, phytohormone function (the crosstalk between phytohormone and the application of phytohormones) and gene identification (such as *CmoNAC1*, *CmHKT1* and *CmCNIH1*) in relation to the responses of pumpkins to salt stress have been reported over the last 10 years [19,20,21,22,23]. Unfortunately, the comparative root morphology and transcriptome under salt stress in *C. moschata* and *C. Maxima* remains unclear, which restricts research on the salt tolerance mechanism of pumpkins.

As the first sensors of salt stress, roots rapidly reduce growth that relies on meristem cell activity [24], in which meristem cell proliferation and differentiation in roots are regulated via coordinating hormonal networks, molecular factors, and environmental factors [25]. Auxin is thought to play a fundamental role in root system architecture by regulating cell division, expansion, and differentiation [26], and salt stress inhibits auxin signaling to reduce root meristem length and cell numbers [24]. Meanwhile, abscisic acid (ABA) induces growth inhibition of roots via crosstalk to auxin [2,27]. By contrast, regarding to cytokinin (CK) signaling, AHK1 (*Arabidopsis* histidine kinase 1), the CK receptor, acts as a positive regulator in response to salt stress [28], and mutation of CK receptor genes (*AHKs* [29], *AHPs* [30], *B-type ARRs* [31]), which indicate enhanced salinity tolerance. Nevertheless, the changes in plant hormone signal pathway in the root under salt stress condition between *C. moschata* and *C. Maxima* is poorly understood.

To uncover the relationship between NaCl concentration and root architecture and explore differentially expressed genes (DEGs) under salt stress in pumpkins, we not only investigated the effect of NaCl concentration on root architecture in *C. moschata* (Cmo-1, Cmo-2 and Cmo-3), *C. Maxima* (Cma-1, Cma-2 and Cma-3), and their hybrids (Ch-1, Ch-2 and Ch-3) at the germination and seedling stages but also examined the changes in transcriptome levels by performing RNA-sequencing (RNA-seq) on the roots of Cmo-3 and Cma-2 in the presence and absence of salt stress. Hence, this study offers insight into the NaCl doses that lead to mild and severe salt stress, thereby improving our understanding of the relationship between root architecture and salt tolerance in pumpkins. Moreover, candidate genes were screened in the plant hormone signaling pathways in this study, which will serve as a valuable reference for future molecular breeding efforts aimed at developing salt-tolerant pumpkin rootstocks.

## 2. Results

### 2.1. Effect of NaCl on the Root Morphology of Pumpkins at the Germination Stage (Experiment 1)

**Figure 1:**
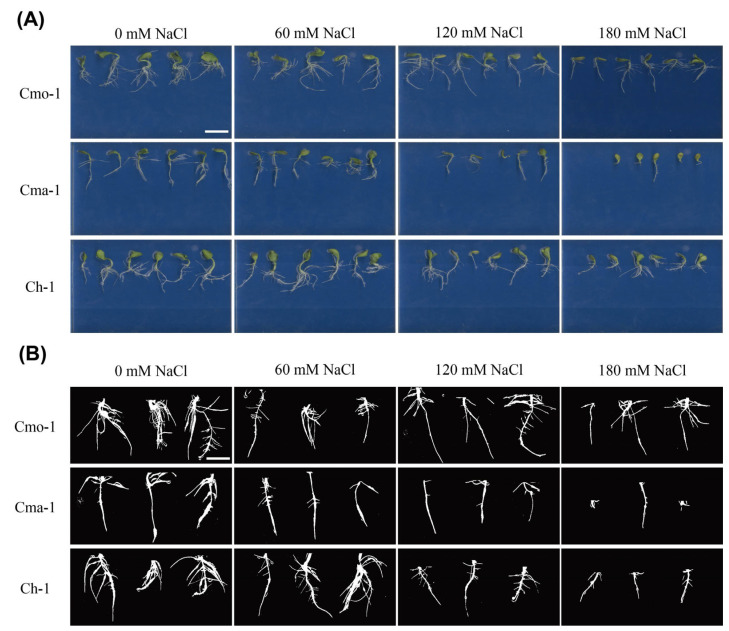
Root morphology under different concentrations of NaCl treatment at the seed germination stage. (**A**) Root morphology after 72 h of 0, 60, 120, and 180 mM NaCl treatment in Cmo-1, Cma-1, and Ch-1. (**B**) Root projection after 72 h of 0, 60, 120, and 180 mM NaCl treatment in Cmo-1, Cma-1, and Ch-1. The scale bars in A and B are 3 cm and 2.4 cm in length, respectively.

As shown in Figure 1A,B, 180 mM NaCl treatment significantly inhibited the growth of root in Cmo-1, Cma-1 and Ch-1. The root morphology under 60 mM NaCl treatment was similar to that under 0 mM NaCl treatment in Cmo-1, Cma-1, and Ch-1 (Figure 1).

**Figure 2:**
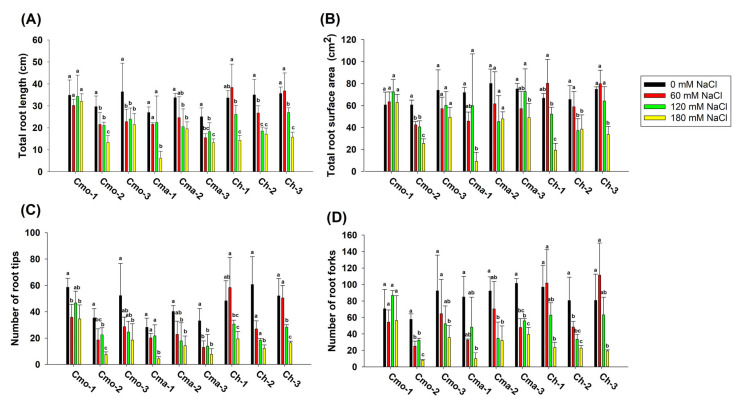
Effect of different concentrations of NaCl on the root morphology index at seed germination stage. (**A**) total root length. (**B**) total root surface area. (**C**) number of root tips. (**D**) number of root forks. Values indicate the mean ± SD (*n* = 9). Different letters above the bars indicate significant differences (*p* ≤ 0.05) according to a least significant difference (LSD) test.

In addition to Ch-1, the effect of the application of 180 mM NaCl on the total root length was significant (*p* value < 0.05) compared to that of the 0 mM NaCl treatment among the eight cultivars (Figure 2A). Compared to that under 0 mM NaCl treatment, the total root length of the Cma-1 and Cma-3 treated with 180 mM NaCl was significantly reduced, by 76.15% and 47.18%, respectively (Figure 2A). Similarly, among the nine pumpkin cultivars, the number of root tips and root forks significantly decreased under 180 mM NaCl treatment compared to that under 0 mM NaCl treatment (Figure 2C,D). However, there was no significant difference in the total root surface area between 0 mM NaCl and 60 mM NaCl treatments among the nine cultivars (Figure 2B). For Cmo-1, Cmo-2, and Cma-3, there was significant difference in the total root length between the 0 mM NaCl treatment (58.55 cm, 35.33 cm, and 33.08 cm, respectively) and 60 mM NaCl treatment (35.77 cm, 18.55 cm, and 13.00 cm, respectively) (Figure 2C). There was no significant difference among the 4 parameters of root morphology between the 60 mM and 120 mM NaCl treatments in Cmo-1, Cmo-2. Cmo-3, Cma-1, Cma-2, and Cma-3 (Figure 2).

The cultivar, NaCl, and interaction between the cultivar and NaCl significantly affected the total root length and the total root surface area. Meanwhile, the cultivar and NaCl, not the interaction of cultivar and NaCl, significantly affected the number of root tips and root forks (Table 1).

### 2.2. Effect of NaCl on the Plant and Root Weight of PUMPKIns at the Seedling Stage (Experiment 2)

**Figure 3:**
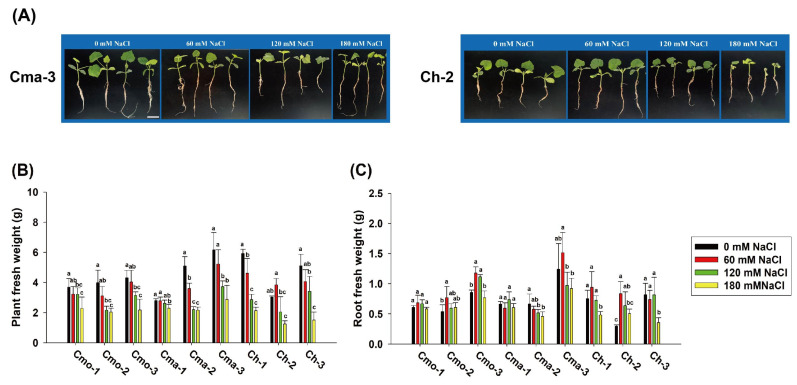
Effect of different concentrations of NaCl on plant and root growth at the seedling stage. (**A**) Seedlings after 4 days of 0, 60, 120, and 180 mM NaCl treatment among Cma-3 and Ch-2. The scale bars in (**A**) are 5 cm in length. (**B**) The effect of different concentrations of NaCl on plant fresh weight at the seedling stage. (**C**) The effect of different concentrations of NaCl on root fresh weight at the seedling stage. Values indicate the mean ± SD (*n* = 9). Different letters above the bars indicate significant differences (*p* ≤ 0.05) according to a least significant difference (LSD) test.

Among the nine cultivars, 180 mM NaCl treatment repressed the growth of plants, better than 0 mM NaCl treatment. Under 180 mM NaCl treatment, Cma-3 plants grew better Ch-2 plants (Figure 3A). Among the nine cultivars, plant fresh weight significantly decreased under 180 mM NaCl treatment compared to that under 0 mM NaCl treatment. Nevertheless, the root fresh weight significantly increased under 60 mM NaCl treatment (0.77 g, 1.17 g, and 0.84 g, respectively) compared to that under 0 mM NaCl treatment (0.53 g, 0.85 g, and 0.30 g, respectively) among Cmo-2, Cmo-3, and Ch-2. The cultivar, NaCl and the interaction between the cultivar and NaCl significantly affected the plant fresh weight and root fresh weight (Table S2).

### 2.3. Effect of NaCl on the Root Morphology of Pumpkins at the Seedling Stage (Experiment 2)

**Figure 4:**
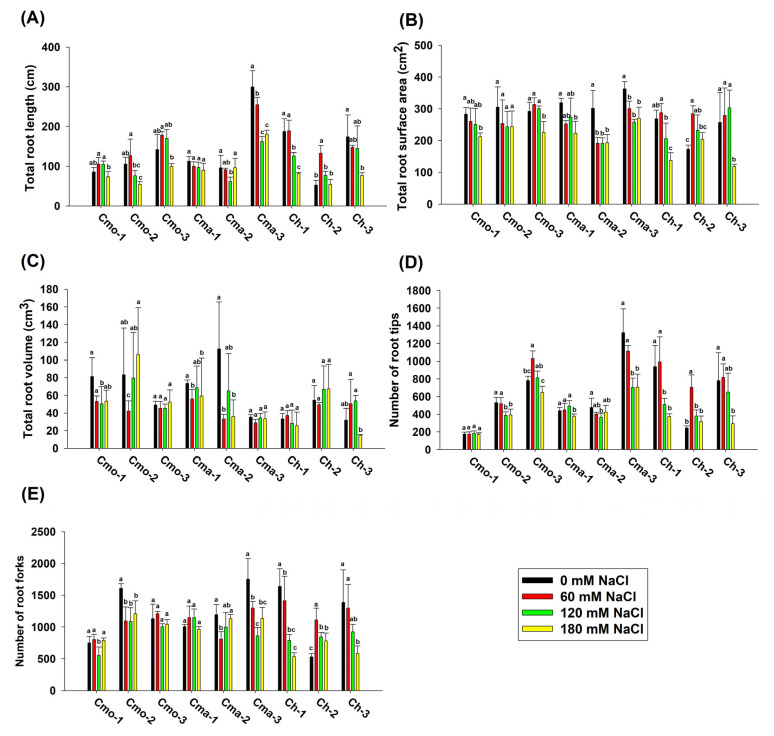
The effect of different concentrations of NaCl on the root morphology index at the seedling stage. (**A**) Total root length. (**B**) Total root surface area. (**C**) Total root volume. (**D**) The number of root tips. (**E**) The number of root forks. Different letters above the bars indicate significant differences (*p* ≤ 0.05) among the plants treated with 0, 60, 120, and 180 mM NaCl. Values indicate the mean ± SD (*n* = 9). Different letters above the bars indicate significant differences (*p* ≤ 0.05) according to a least significant difference (LSD) test.

In addition to Ch-2, the total root length and total root surface area were decreased under 180 mM NaCl treatment compared to that under 0 mM NaCl treatment (Figure 4A,B). Under 180 mM NaCl treatment conditions, the total root length was the highest in Cma-3 (180.58 cm) and the lowest in Ch-2 (54.30 cm). In addition, the total root surface area was the highest in Cma-3 (270.88 cm^2^) and the lowest in Ch-3 (118.61 cm^2^) among the nine cultivars (Figure 4A,B). The total root volume changed irregularly among the nine cultivars, with significant differences in total root volume between 0 and 60 mM NaCl treatments in Cma-1 and Cma-2 (Figure 4C).

The effect of applying 180 mM NaCl on the number of root tips was significant (*p* value < 0.05) compared to that under 0 mM NaCl treatment in Cmo-2, Cma-1, Cma-3, Ch-1, and Ch-3 (Figure 4D). Compared to the results under 0 mM NaCl treatment, the number of root tips in Cmo-2 and Ch-3 treated with 180 mM NaCl was significantly reduced by 26.04% and 62.32%, respectively (Figure 4D). Under different concentration of NaCl treatment, the number of root tips of Cma-3 was the highest (Figure 4D). Similarly, the number of root forks in Cmo-2, Cma-3, Ch-1, and Ch-3 significantly decreased under 180 mM NaCl treatment compared to that under 0 mM NaCl treatment.

The cultivar, NaCl and the interaction between the cultivar and NaCl significantly affected the total root length, total root surface area, number of root tips, and number of root forks (Table 2). Only the cultivar significantly affected the total root volume (Table 2).

### 2.4. Transcriptomic Differential Profiles of Roots Induced by Salt Stress (Experiment 3)

#### 2.4.1. Identifying DEGs Involved in Salt Stress

**Figure 5:**
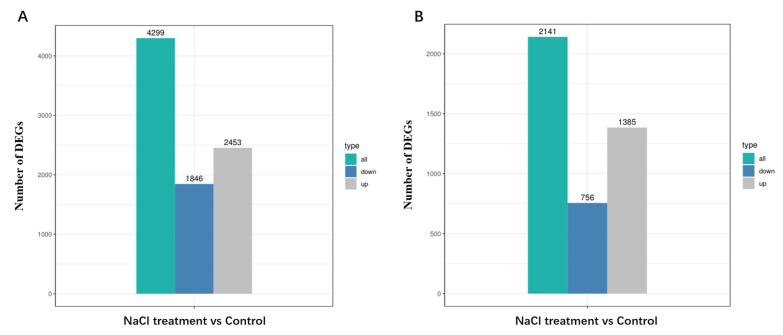
The number of differentially expressed genes (DEGs) identified through a pairwise comparison (NaCl vs. control) in the roots. (**A**) DEGs in Cmo-3. (**B**) DEGs in Cma-2.

We observed a total of 4299 DEGs in Cmo-3 and 2141 DEGs in Cma-2 under salt stress (Figure 5). In total, 2453 genes were upregulated, and 1846 genes were downregulated when comparing the NaCl treatment to the control in Cmo-3 (Figure 5A). Likewise, 1385 genes were upregulated, and 756 genes were downregulated when comparing NaCl treatment to the control in Cma-2 (Figure 5B). These findings suggest that there are differences in gene expression between *C. moschata* and *C. maxima* under salt stress.

#### 2.4.2. GO Enrichment Analysis

**Figure 6:**
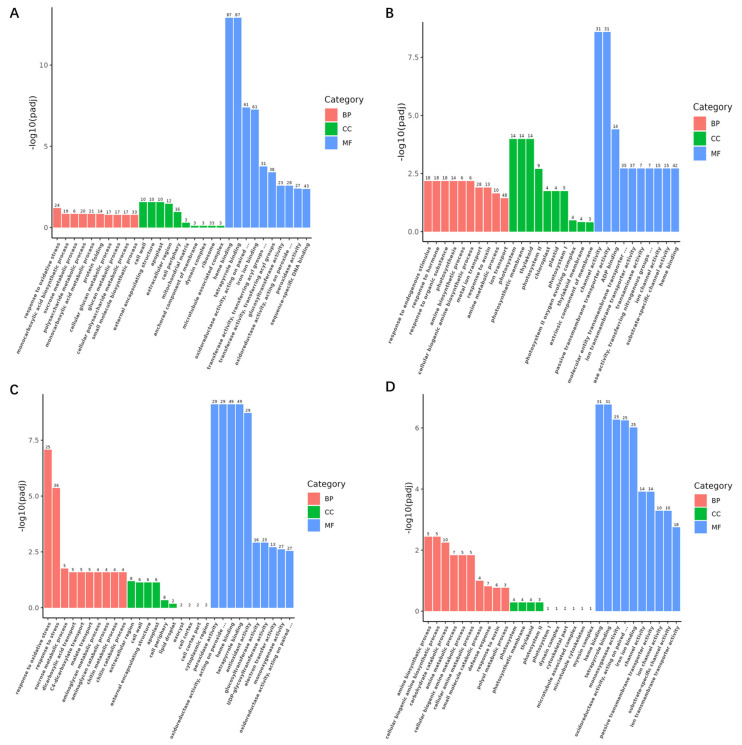
GO terms enriched with DEGs involved in roots under salt stress. (**A**) GO terms enriched with up-regulated DEGs in the roots of Cmo-3. (**B**) GO terms enriched with down-regulated DEGs in the roots of Cmo-3. (**C**) GO terms enriched with up-regulated DEGs in the roots of Cma-2. (**D**) GO terms enriched with down-regulated DEGs in the roots of Cma-2. The red, green and blue colors indicate biological processes (BP), cellular components (CC), and molecular function (MF), respectively.

Subsequently, we conducted GO (Gene Ontology) analysis on the DEGs of Cmo-3 and Cma-2 under salt treatment. To explore the specific pathways with GO enrichment, the top 10 GO terms were analyzed for the biological process (BP), cellular component (CC), and molecular function (MF) components (Figure 6) based an adjusted *p*-value < 0.05. In Cmo-3, a large number of up-regulated genes were found to be associated with tetrapyrrole binding (87 DEGs), heme binding (87 DEGs), iron ion binding (61 DEGs) and oxidoreductase activity, acting on paired donors, with the incorporation or reduction in molecular oxygen (61 DEGs) (Figure 6A). The important GO terms enriched with down-regulated genes included channel activity, passive transmembrane transporter activity, ion transport, and heme binding (Figure 6B). In Cma-2, the response to the 120 mM NaCl, response to oxidative stress (25 DEGs), response to stress (36 DEGs), peroxidase activity (29 DEGs), heme binding (49 DEGs), tetrapyrrole binding (49 DEGs), antioxidant activity (29 DEGs), oxidoreductase activity, and acting on peroxide as acceptor (29 DEGs) were the significant GO terms enriched by up-regulated genes (Figure 6C). Five GO terms (iron ion binding, tetrapyrrole binding, heme binding, monooxygenase activity, and oxidoreductase activity, acting on paired donors, with incorporation or reduction in molecular oxygen) were enriched by down-regulated genes (Figure 6D). GO terms enriched with up-regulated genes were different to GO terms enriched with down-regulated genes in the same species under salinity stress conditions (Figure 6).

#### 2.4.3. KEGG Enrichment Analysis

**Figure 7:**
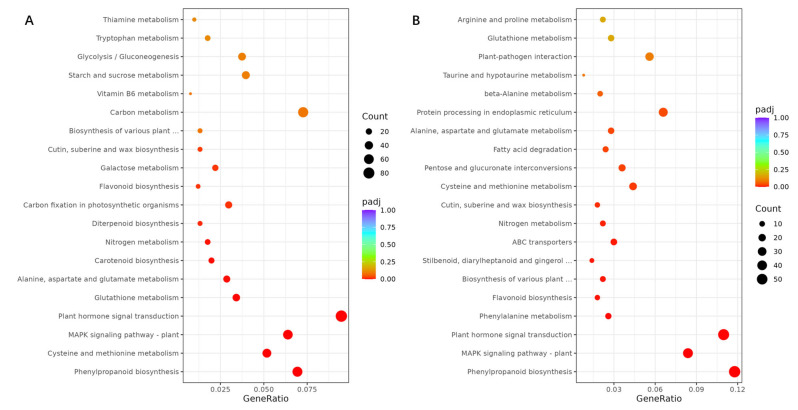
KEGG enrichment analysis of DEGs unique in the roots of Cmo-3 (**A**) and Cma-2 (**B**) under salt stress.

The significantly enriched KEGG (Kyoto Encyclopedia of Genes and Genomes) pathways of roots in Cmo-3 and Cma-2 under salt stress were also analyzed (Figure 7). Plant hormone signal transduction, Phenylpropanoid biosynthesis and the MAPK signaling pathway, as the most significant KEGG pathways, were enriched with 86, 63, and 58 DEGs, respectively, in Cmo-3 (Figure 7A) and 55, 59, and 42 DEGs, respectively, in Cma-2 (Figure 7B). Regarding the metabolism pathways, in Cmo-3 and Cma-2, 18 and 8 DEGs corresponded to tryptophan metabolism, 10 and 7 DEGs to zeatin biosynthesis, 18 and 8 DEGs to carotenoid biosynthesis and 36 and 17 DEGs to starch and sucrose metabolism, respectively. The relationship between the ABA pathway and the stress-induced MAPK signaling pathways is very close and needs to be further studied.

#### 2.4.4. Identification of DEGs Involved Plant Hormone Signaling Transduction

**Figure 8:**
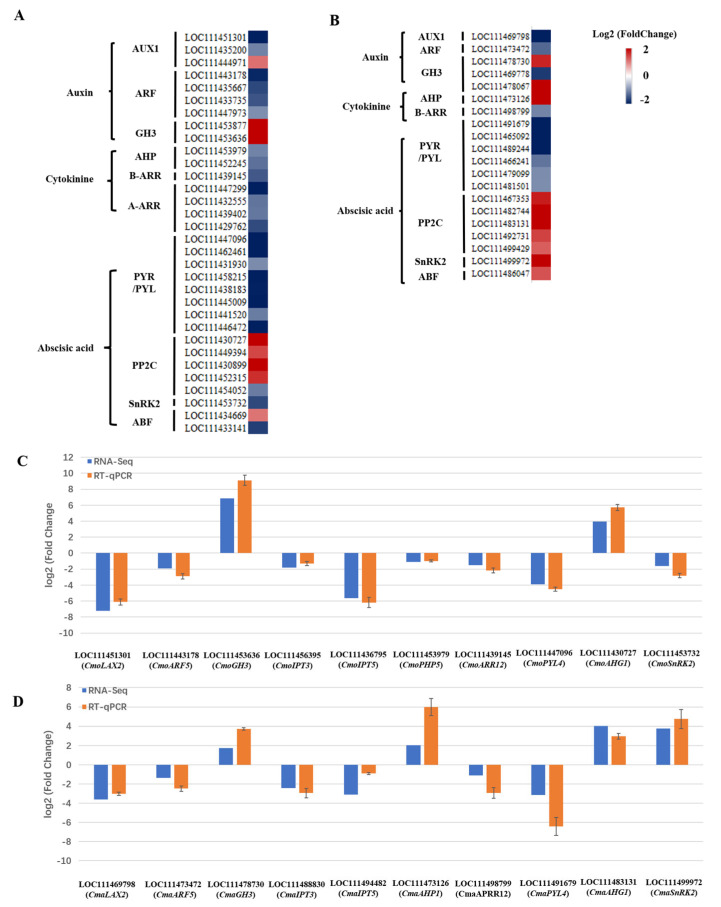
DEGs involved in the auxin, cytokinin and abscisic acid signaling pathway. Expression changes in the DEGs in Cmo-3 (**A**) and (**B**) by RNA-seq. The relative expression of key genes using RT-qPCR in Cmo-3 (**C**) and Cma-2 (**D**).

We observed changes in the auxin, cytokinin, and abscisic acid signaling pathways in the roots of Cmo-3 and Cma-2 under salt stress. As shown in Figure 8, there was a downregulation of *ARF* (*auxin response factor*), *B-ARR* (*type-B response regulator*), and *PYR* (*pyrabactin resistance*)/*PYL* (*PYR-LIKE*) genes in Cmo-3 and Cma-2 under NaCl treatment, compared to the control, which indicates that the cytokinin signaling pathway was negatively regulated under salt stress. Similarly, expression of more *AUX1* (*auxin resistant 1*) genes was also downregulated by NaCl treatment. Conversely, the expression of more *GH3* (*gretchen hagen 3*) and *PP2C* (*2C type protein phosphatases*) genes was upregulated in the NaCl treatment vs. the control. However, the expression of *SnRK2* (*sucrose non-fermenting-1-related protein kinase 2*) genes, *ABF* (*abscisic acid-responsive transcription factors*) and *AHP* (*histidine-containing phosphotransmitter*) genes was downregulated in Cmo-3 and upregulated in Cma-2 under NaCl treatment, compared to the control. In Cmo-3 and Cma-2, 10 DEGs were selected for RT-qPCR trials. As the key regulatory enzyme of cytokinin biosynthesis, *IPT3* (*isopentenyl transferase 3*) and *IPT5* (*isopentenyl transferase 5*) expression was sharply downregulated under NaCl treatment. As expected, the results of the RT-qPCR were similar to the transcriptome data (Figure 8C,D).

## 3. Discussion

### 3.1. Changes in the Root Architecture Under Mild and Severe Salt Stress Conditions

#### 3.1.1. Total Root Length

**Figure 9:**
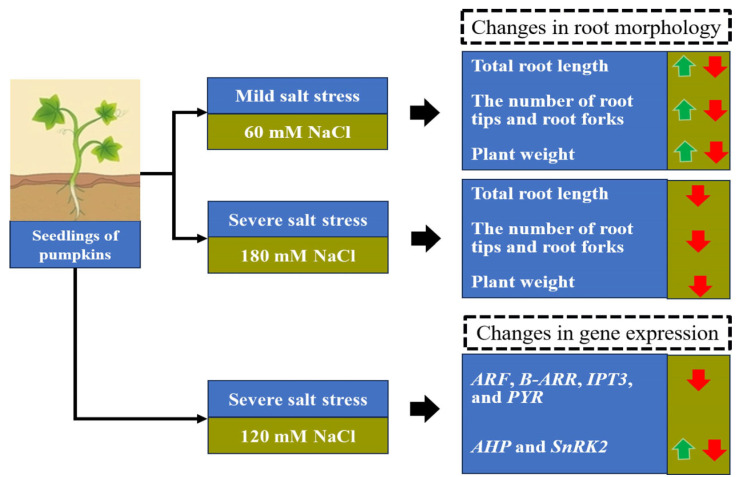
A working model of root in response to salt stress in the pumpkin. Symbols: red arrows indicate upregulation, and green arrows indicate downregulation.

Under saline conditions, plants exhibit root phenotypic plasticity by dynamically modulating both root architecture parameters and directional growth [32,33]. Previous studies have shown that the application of 50 to 200 mM NaCl inhibits root length in wheat [7], while the application of 75 to 150 mM NaCl inhibits both primary root and lateral root growth [34]. As expected, a high concentration of NaCl (180 mM NaCl) caused a decrease in total root length in this study. Moreover, 60 mM NaCl treatment caused a significant decrease in total root length in four cultivars (Cmo-2, Cmo-3, Cma-3, and Ch-2) at the germination stage and in two cultivars (Cma-3 and Ch-2) at the seedling stage in this study, revealing natural variation in balancing growth between the primary and lateral roots. Therefore, we suggest that a solution with a concentration of NaCl is equal to or greater than 120 mM is the suitable dose for research on root growth in response to salt stress (Figure 9). Meanwhile, a 60 mM NaCl solution corresponds to mild salt stress in pumpkins, as also found in previous studies [21,23].

On the other hand, under mild salt stress (60 mM NaCl) conditions, our results show that total root length and the number of root tips increased at the seedling stage in the four cultivars compared to the results under 0 mM treatment (Figure 3A), which is contrary to the previous studies [35,36]. Similarly, 150 mM NaCl led to an increase in root length in UDEC9 and BO78 (*Chenopodium quinoa* Willd.) [12]. Hence, the root length of pumpkin can decrease or increase under low salt stress, dependent on the genotypes of the pumpkin in question.

#### 3.1.2. Lateral Root Initiation

Lateral root development is partially blocked by salt stress [34,37]. Similarly, the application of 180 mM NaCl caused a significant decrease in the number of root tips among the five cultivars (Cmo-2, Cma-1, Cma-3, Ch-1, and Ch-3) at the seedling stage in this study (Figure 4A). Nevertheless, under treatment with a high concentration of NaCl (180 mM), the number of root tips and root forks significantly decreased in all cultivars at the germination stage in this study. We hypothesized that the response of root to salt stress at the germination stage would be stronger than that at the seedling stage in pumpkin.

#### 3.1.3. Salt-Tolerant Pumpkin

Additionally, Cma-3, as a salt-tolerant material, was screened by cluster analysis and multiple comparison in this study (Figure S1). Total root length and number of root tips in the seedlings of Cma-3 were the highest among the nine cultivars under 180 mM NaCl treatment, which is similar to the results of other crops in previous studies [5,38]. It would be worth exploring the physiological salt resistance mechanism of Cma-3, which may play a key role in the future utilization and production of germplasm resources.

### 3.2. Changes in Gene Expression in the Roots of C. Moschata and C. Maxima

#### 3.2.1. The Number and Function of DEGs

RNA sequencing (RNA-seq) has been widely used in *C*. *moschata* and *C. maxima* under chilling stress, cold stress, powdery mildew infection and sex differentiation conditions [39,40,41,42,43], in which more than 1000 DEGs were found, and the plant hormone signaling pathway played an important role. In this study, we found more than 50 DEGs enriched in the plant hormone signal transduction pathway of KEGG in Cmo-3 and Cma-2, which significantly affects root growth and differentiation [25]. Even though the salt tolerance of Cmo-3 and Cma-2 is the same, the expression of the *AHP*, *SnRK2*, and *ABF* genes in the hormone signaling pathways is different between Cmo-3 and Cma-2. The different transcriptome profiles under salt stress may result in the same phenotype in pumpkins with the *AHP*, *SnRK2*, and *ABF* representing potential key genes in terms of salt tolerance and root development based on previous studies [29,44].

#### 3.2.2. Auxin and Cytokinin Signaling Pathway

Severe salt stress (more than 120 mM NaCl) inhibits root growth and lateral root differentiation in this study. Auxin and cytokinin (CK) control a plethora of developmental processes including root development [26,45]. Interestingly, *Arabidopsis gh3oct* mutants [46], *ahp2,3,5* and *arr1,10,12* triple mutants were reported to be salt tolerant [30], which indicates that the auxin and CK signaling pathways negatively regulate salt tolerance in *Arabidopsis*. Our results showed that NaCl treatment led to a decrease in the expression of *ARF, B-ARR and A-ARR (only in Cmo-3)* genes. These results suggest that *B-ARR genes* also negatively regulate salt tolerance in pumpkin. Salt stress could repress auxin signaling to reduce root meristem growth [24], while the expression of most genes of the auxin signal pathway was downregulated under salt stress in this study (Figure 8). Nevertheless, the expression of *GH3* genes was sharply increased under NaCl treatment in this study, indicating *GH3* genes may be a positive regulator in response to NaCl treatment in pumpkins.

#### 3.2.3. Abscisic Acid Signaling Pathway

Abscisic acid (ABA) is especially important for plant adaptation to abiotic stress [47]. Additionally, the cross of ABA and auxin promotes the lateral root quiescence of *Arabidopsis* seedlings under salt stress [27,48]. We found salt stress increased the expression of *PP2C genes* and decreased the expression of *PYR/PYL* genes. Previous studies showed that the overexpression of *RCAR6/PYL12* could increase water use efficiency with high growth rates, while the overexpression of *GhPYL10/12/26* in *Arabidopsis* could increase root growth under drought stress, compared to the wild type [49]. It is suspected that salt stress decreases the expression of *PYR/PYL* genes to inhibit root growth and lateral root differentiation in both Cmo-3 and Cma-2.

#### 3.2.4. Sucrose Non-Fermenting-1-Related Protein Kinase 2 (SnRK2)

Sucrose non-fermenting-1-related protein kinase 2 (SnRK2) tightly modulate plant growth and stress responses [4,50]. Moreover, the expression of *SnRK2* family genes is induced by ABA, drought, and other stresses in different crop [51,52,53,54]. On the one hand, the overexpression of *ZmSnRK2.11* may reduce salt tolerance in *Arabidopsis* [55]; additionally, *snrk2.4* knockout mutants displayed a reduced primary root length [56]. On the other hand, the overexpression of *CsSnRK2.5* was found to increase tolerance to drought stress in *Arabidopsis* [57]. The functions of different *SnRK2* genes are different, which is similar to our results. In this study, we found salt stress to increase the expression of *SnRK2* in Cma-2 and decrease the expression of *SnRK2* in Cmo-3, which indicates that *SnRK2* is a positive regulator in Cma-2 and a negative regulator in Cmo-3 under NaCl treatment. In this way, we analyzed the function of SnRK2 in pumpkins in response to salt stress.

## 4. Materials and Methods

### 4.1. Plant Materials

As Shown in Table S1, 9 pumpkin cultivars (Cmo-1, Cmo-2, Cmo-3, Cma-1, Cma-2, Cma-3, Ch-1, Ch-2 and Ch-3) were randomly selected from the pumpkin germplasm nursery of the institute of coastal agriculture at the Hebei academy of agriculture and forestry sciences, based on their genetic backgrounds, inbred lines, hybrids and product uses. Cmo-1, Cmo-2 and Cmo-3 are *C. moschata*; Cma-1, Cma-2 and Cma-3 are *C. maxima*; Ch-1, Ch-2 and Ch-3 are hybrids of *C. moschata* and *C. maxima*.

### 4.2. Experiment 1 (Effect of NaCl on Root Morphology of Pumpkins at the Germination Stage)

In experiment 1, the seeds were soaked in ultrapure water at 50 °C for 30 min and the seed coats were removed. Then, the seeds were soaked in 70% alcohol for 40 s, washed with sterile water 3 times and finally placed on an Murashige and Skoog (MS) medium in the dark at 27 °C for 72 h. Uniform germination seeds were then selected and transferred to new MS medium containing 0, 60, 120, and 180 mM NaCl at 25 ± 2 °C under cool-white fluorescent lighting with a 16 h photoperiod using a light intensity of 40 µmol m^−2^ s^−1^, as shown in Figure S2. There was a total of four treatments: (1) 0 mM NaCl, (2) 60 mM NaCl, (3) 120 mM NaCl, and (4) 180 mM NaCl. Each treatment consisted of 3–5 germination seeds per replication. Experiment 1 was replicated three times, and a total of 60 germination seeds were used for a single cultivar. All samples were collected after 72 h of NaCl treatment to measure the root morphological parameters (Figure S2).

### 4.3. Experiment 2 (Effect of NaCl on Root Morphology of Pumpkins at Seedling Stage)

Experiment 2 was carried out in seedling culture room, institute of coastal agriculture, at the Hebei academy of agriculture and forestry sciences, China. The seeds were sowed into plastic plugs with a “Jiahui” substrate (Liaocheng, China) which contained 20–25% organic matter and 8–10% humic acid. After 15 days of growth (one leaf and two cotyledon stage), young plants were transferred to some plastic boxes (length 46 cm × width 32 cm × depth 12 cm) with Hoagland nutrient solution (Hunan Hoagland Biological Engineering Co., Ltd., Changsha, China) containing 0, 60, 120, and 180 mM NaCl, as shown in Figure S1. During the growth season of pumpkin, the temperature at (28 ± 2) °C/(16 ± 2) °C (day/night) was maintained. There was a total of four treatments: (1) 0 mM NaCl, (2) 60 mM NaCl, (3) 120 mM NaCl, and (4) 180 mM NaCl. Each treatment utilized 4 plants per replication. Experiment 2 was replicated three times, and a total of 48 plants were used for a single cultivar. Samples were collected after 72 h of NaCl treatment to measure the root morphological parameters (Figure S3). Meanwhile, the roots of Cmo-3 and Cma-2 under 0 mM NaCl and 120 mM NaCl treatment were immediately frozen in liquid nitrogen and stored at −80 °C for RNA-seq.

### 4.4. Measurement of Root Morphological Parameter

The samples separated as mentioned above were scanned with an Epson Perfection V700 scanner to obtain a grayscale TIFF image. This image was then analyzed with the WinRHIZO Pro image processing system (Regent Instruments Inc., 2672 Chemin Sainte-Foy, Quebec City, QC G1V1V4, Canada) to obtain total root length, total root surface area, total root volume, number of root tips and number of root forks.

### 4.5. Experiment3 (RNA-Seq Analysis of Roots of Cmo-3 and Cma-2 Under NaCl Treatment)

#### 4.5.1. Experiment Design

Based on the results of experiment 1 and 2 in this study, Cmo-3 and Cma-2 have moderate salt tolerance and were selected in experiment 3. We also used 120 mM NaCl (severe salt stress) and 0 mM NaCl treatment. Seedlings cultivation was performed as in experiment 2. After 2 days of NaCl treatment, the roots were collected for RNA-seq analysis.

#### 4.5.2. RNA Extraction, Library Construction and Sequencing

Total RNA was extracted from the root samples with an Omega Plant RNA kit (Omega Bio-tek, Cat. No. R682701, Norcross, GA, USA) and used to make RNA-seq libraries with the Illumina TruSeq RNA Kit (NEB, Cat. No. E7530, Ipswich, MA, USA) following the manufacturer’s instructions. The libraries were sequenced using the Illumina NovaSeq X plus platform in the 150 bp paired-end mode. Three biological replicates were performed for the RNA-seq experiment.

#### 4.5.3. RNA-Seq Analysis

Low-quality reads were removed based on the conditions of containing only adaptors, with unknown nucleotides > 5%, or a Q20 score < 20%. Clean reads were mapped to the perennial ryegrass genome (https://ryegrassgenome.ghpc.au.dk/ (accessed on 4 April 2025)) [58], using HISAT2-2.1.0 with default settings [59]. The feature counts was used to obtain raw counts [60], and reads per kilobase of exon per million reads (RPKM) were calculated to measure the expression levels of the genes. Differentially expressed genes (DEGs) were identified when the criteria of a |log2 Fold Change| > 1 and an adjusted *p*-value < 0.05 were met. DEGs related to metabolism were identified based on the Kyoto Encyclopedia of Genes and Genomes (KEGG) (www.kegg.jp (accessed on 5 April 2025)).

#### 4.5.4. RT-qPCR

RT-qPCR was performed as described in Xue.S. et al. (2024) [42] and Wang.Y. et al. (2019) [41]. For the relative quantification of gene expression, *CmoActin* and *CmaActin* were used as an endogenous reference. The primers used are listed in Supplementary Table S3. The relative gene expression levels between the control and salinity stress treatment were calculated using the 2^−ΔΔCt^ method [61].

### 4.6. Statistical Analysis

The data in experiment 1, 2 and 3 were analyzed using a two-way variance test (ANOVA) as a 9 × 4 (cultivar × NaCl) factorial structure, with the SAS 9.4 Software (SAS Institute Inc., Cary, NC, USA). Mean separations among treatments were performed using Student’s *t*-test or the least significant difference (LSD) test at *p* < 0.05. All graphs were plotted using the Sigma Plot 10.0 Software. The relationships between treatments and plant characteristics were determined in Pearson’s correlations (within 5% and 1% error limits).

## 5. Conclusions

Our study provided an integrated view of the morphological and molecular responses in pumpkin exposed to salt stress at the germination and seedling stages. Our data revealed that the total root length and the number of root tips decreased under 180 mM NaCl conditions compared to those under the 0 mM NaCl conditions. Salt stress was considered mild when treated with 60 mM NaCl, and may be considered severe when treated with more than 120 mM NaCl; these findings can be used to evaluate the salt tolerance of the germplasm resources and breeding of salt-tolerant varieties of pumpkins (Figure 9). Using RNA-seq, 4299 DEGs in Cmo-3 and 2141 DEGs in Cma-2 were screened, with plant hormone signal transduction, Phenylpropanoid biosynthesis, and the MAPK signaling pathway found to be the significant KEGG pathways of pumpkin. Altogether, our results help elucidate the mechanisms underlying response to salt stress in pumpkin, providing candidate genes for further studies aimed in increasing tolerance under stress conditions. In the future, the application of plant hormones and an analysis of the functions of genes related to hormone signal transduction pathways should be further explored, which will facilitate the breeding of salt-tolerant pumpkins.

## Figures and Tables

**Table 1:** Results for two-way ANOVA regarding the effects of the cultivar, NaCl, and cultivar × NaCl interaction on root phenotypic parameters at the germination stage.

Experimental Factors	Total Root Length		Total Root Surface Area		Number of Root Tips		Number of Root Forks	
	*F* Value	*p*	*F* Value	*p*	*F* Value	*p*	*F* Value	*p*
Cultivar	9.07	<0.0001	4.16	0.0004	9.54	<0.0001	4.48	0.0002
NaCl	37.03	<0.0001	23.79	<0.0001	39.43	<0.0001	25.94	<0.0001
Cultivar × NaCl	1.91	0.0191	1.89	0.0205	1.48	0.103	1.44	0.121

**Table 2:** Results for the two-way ANOVA regarding the effects of cultivar, NaCl and cultivar × NaCl interaction on root phenotypic parameters at the seedling stage.

Experimental Factors	Total Root Length		Total Root Surface Area		Total Root Volume		Number of Root Tips		Number of Root Forks	
	*F* Value	*p*	*F* Value	*p*	*F* Value	*p*	*F* Value	*p*	*F* Value	*p*
Cultivar	51.69	<0.0001	5.12	<0.0001	2.64	0.0131	48.82	<0.0001	10.58	<0.0001
NaCl	38.38	<0.0001	21.64	<0.0001	0.45	0.7202	30.14	<0.0001	19.19	<0.0001
Cultivar × NaCl	5.08	<0.0001	3.11	0.0001	1.44	0.1168	4.82	<0.0001	5.37	<0.0001

## Data Availability

The raw sequence data reported in this paper have been deposited in the Genome Sequence Archive (Genomics, Proteomics & Bioinformatics 2021) in the National Genomics Data Center (Nucleic Acids Res 2024), China National Center for Bioinformation/Beijing Institute of Genomics, Chinese Academy of Sciences (GSA: CRA025075), and are publicly accessible at https://ngdc.cncb.ac.cn/gsa accessed on 31 December 2025.

## Supplementary Materials

The following supporting information can be downloaded at: https://www.mdpi.com/article/10.3390/plants14111674/s1, Figure S1: The dendrogram of clusters for 9 pumpkin cultivars; Figure S2: Seedlings picture before NaCl treatment; Figure S3: Sample collection under with 0, 60, 120 and 180 mM NaCl treatment; Table S1: pumpkin cultivars information in this study; Table S2: Results for two-way ANOVA regarding the effects of cultivar, NaCl and cultivar * NaCl interaction on plant fresh weight and root fresh weight at seedlings stage; Table S3: Primer sequence information used in RT-qPCR experiment.

## Abbreviations

*A-ARR*	*type-A response regulator*
ABA	Abscisic acid
*ABF*	*abscisic acid-responsive transcription factors*
*AHP*	histidine-containing phosphotransmitter
*AUX1*	*auxin resistant 1*
*B-ARR*	*type-B response regulator*
*C. maxima*	*Cucurbita maxima*
*C. moschata*	*Cucurbita moschata*
CK	Cytokinin
DEGs	differential expression genes
GO	Gene Ontology
*GH3*	*gretchen hagen 3*
*IPT3*	*isopentenyl transferase 3*
*IPT5*	*isopentenyl transferase 5*
KEGG	Kyoto Encyclopedia of Genes and Genomes
*PYR*	*pyrabactin resistance*
*PYL*	*PYR-LIKE*
*PP2C*	*2C type protein phosphatases*
RNA-seq	RNA sequencing
*SnRK2*	*sucrose non-fermenting-1-related protein kinase 2*
