# Supplementary material for: Salt Stress Leads to Morphological and Transcriptional Changes in Roots of Pumpkins (Cucurbita spp.)
Source: Plants (Basel). 2025 May 30;14(11):1674. doi: 10.3390/plants14111674 (PMC12157933; doi:10.3390/plants14111674)
Supplement: Supplementary file 1 [file plants-14-01674-s001.zip › plants-3640731-supplementary.pdf]

Table S1 pumpkin cultivars information in this study

Cultivar name	Latin Name	Characteristic	Use
Cmo-1	*Cucurbita moschata*	inbred lines	food
Cmo-2	*Cucurbita moschata*	inbred lines	food
Cmo-3	*Cucurbita moschata*	hybridization	rootstock
Cma-1	*Cucurbita maxima*	hybridization	food
Cma-2	*Cucurbita maxima*	hybridization	rootstock
Cma-3	*Cucurbita maxima*	hybridization	rootstock
Ch-1	hybrids of *Cucurbita moschata* and *Cucurbita maxima*	hybridization	rootstock
Ch-2	hybrids of *Cucurbita moschata* and *Cucurbita maxima*	hybridization	rootstock
Ch-3	hybrids of *Cucurbita moschata* and *Cucurbita maxima*	hybridization	rootstock

Table S2 Results for two-way ANOVA regarding the effects of cultivar, NaCl and cultivar × NaCl interaction on plant fresh weight and root fresh weight at seedlings stage

Experimental factors	Plant fresh weight		Root fresh weight	
	F value	*P*	F value	*P*
Cultivar	9.01	<.0001	13	<.0001
NaCl	84.69	<.0001	16.73	<.0001
Cultivar × NaCl	4.02	<.0001	3.11	0.0003

Table S3 Primer sequence information used in RT-qPCR experiment

Gene name	Forward primer (5’-3’)	Reverse primer (5’-3’)
LOC111451301 (*CmoLAX2*)	TGTTCACCTTCAAATCCGCT	GAAGAGGAGGTGGGCACTGAT
LOC111443178 (*CmoARF5*)	CGTTAGCCTTCACGGAGC	GATGAATTTGGGTTTATGGGA
LOC111453636 (*CmoGH3.1*)	ATCTTTTCCTCTCATCCCATTTC	TCTTGGTTTCAGCCTTCACG
LOC111456395 (*CmoIPT3*)	CGAACTTTCAATCGAGATATG	TCCCTTAGCTACCATTTTGTC
LOC111436795 (*CmoIPT5*)	TGGACAACGGCTTCATTAAC	ATCCTCGTTTGCTCCGAA
LOC111453979 (*CmoPHP5*)	TTGAGCAGGGTTATCTTGAT	CGAATCATTGTAGAATAATGAC
LOC111439145 (*CmoARR12*)	CATTTGGGAGTAAAGACGGT	CTGTATCAGTCCAGAAGAGGTG
LOC111447096 (*CmoPYL4*)	AACTACCGATCCGTTACCAC	AGCGACATGAGCGAGAGAT
LOC111430727 (*CmoAHG1*)	CTACTACGATAGACCTCAACGC	CCAAAGCTAGACGAGTCAGC
LOC111453732 (*CmoSnRK2*)	TTTGGTGTTGCGAAACTTGT	CATATTCCATGACGATTGCC
CmoActin	AGCCATCTCTCATCGGTAT	CATGGTTGAACCACCACTG
LOC111469798 (*CmaLAX2*)	CACCGTCTATATCATCCCTG	TGTCGATTTGTCGTATAAAGTT
LOC111473472 (*CmaARF5*)	AGAACTACGACGAACTGTGC	CTTGACTTCTGAGGGTGACA
LOC111478730 (*CmaGH3*)	AGGTGACAGGGTTCCACA	AAGAGATGACGCGTTCTCTA
LOC111488830 (*CmaIPT3*)	TCGTTCTTAAACGAGGAAAC	TCTGCTTCTTTGCCTCG
LOC111494482 (*CmaIPT5*)	TGGACAACGGCTTCATTA	GGTGGAGATTCCATCCTC
LOC111473126 (*CmaAHP1*)	GATGCTTATGTTCACCAGTT	CATTTCAAACACCCTTCAA
LOC111498799 (*CmaAPRR12*)	CCGAAGGCAAAGAACAA	GCACTGTCTGCATTACTGG
LOC111491679 (*CmaPYL4*)	CAGGTCCAAGTCGTCTCC	GGATTGGTAGTTTCGCAG
LOC111483131 (*CmaAHG1*)	TTGTAATCTCCAAACCAGAAG	ACTGATGTTGTCTTTGCTTC
LOC111499972 (*CmaSnRK2*)	GGAAGATTGCTGATGTTTG	GTCCGACTCCAAATCCT
CmaActin	CCTCTCAATCCCAAAGCTAACAG	CGGCCTGGATAGCAACATACA

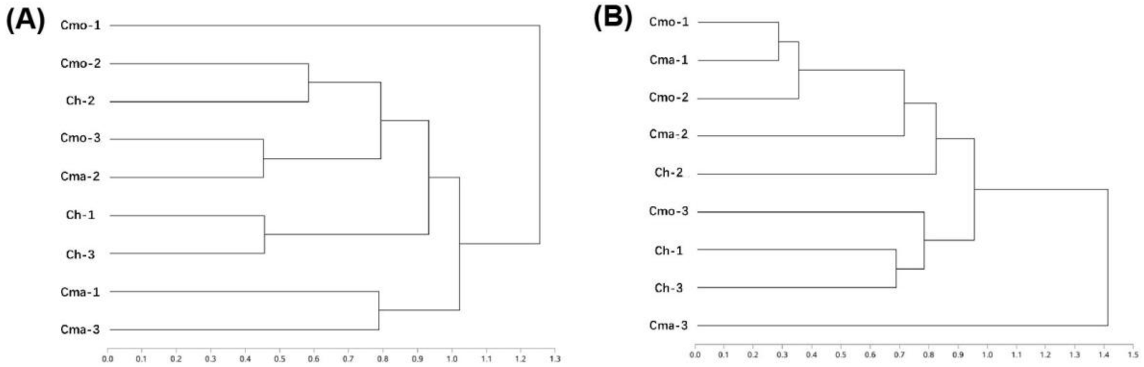

Figure S1 The dendrogram of clusters for 9 pumpkin cultivars. (A) seed germination stage. (B)seedling stage.

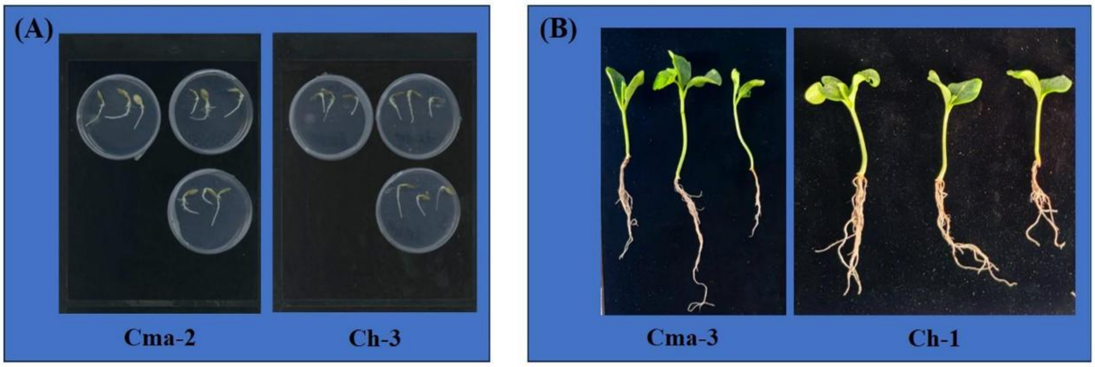

Figure S2 Seedlings picture before NaCl treatment. (A) Cma-2 and Ch-3 at seed germination stage. (B) Cma-3 and Ch-1 at seedling stage.

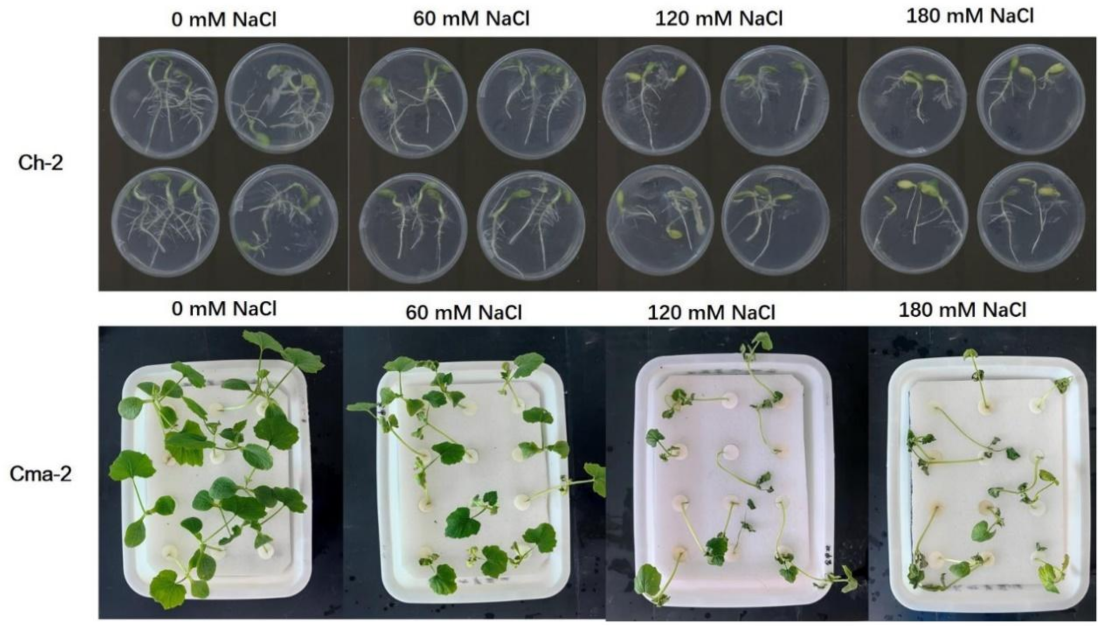

Figure S3 Sample collection under with 0, 60, 120 and 180 mM NaCl treatment. (A) sample collection after 72 hours of NaCl treatment in Ch-2 at seed germination stage. (B) sample collection after 96 hours of NaCl treatment in Cma-2 at seedling stage.
